# Downregulation of heat shock protein 70 protects rheumatoid arthritis fibroblast-like synoviocytes from nitric oxide-induced apoptosis

**DOI:** 10.1186/ar2797

**Published:** 2009-08-27

**Authors:** Eun Ha Kang, Dong Jo Kim, Eun Young Lee, Yun Jong Lee, Eun Bong Lee, Yeong Wook Song

**Affiliations:** 1 Division of Rheumatology, Department of Internal Medicine, Rheumatism Research Institute, Seoul National University Hospital, 28 Yongun-dong, Chongno-gu, Seoul, 110-744, Republic of Korea; 2 Department of Biochemistry, Seoul National University College of Medicine, 28 Yongun-dong, Chongno-gu, Seoul, 110-799, Republic of Korea

## Abstract

**Introduction:**

Heat shock protein 70 (Hsp70) is a well-known anti-apoptotic protein that blocks multiple steps in the stress-induced apoptotic pathway. Enhanced Hsp70 expression has previously been demonstrated in rheumatoid arthritis (RA) fibroblast-like synoviocytes (FLSs). The authors investigated the role of Hsp70 in the survival of RA FLSs in a sodium nitroprusside (SNP)-treated environment.

**Methods:**

Targeted knock-down of Hsp70 was performed by RNA interference in RA FLSs at passage 3-7. After SNP treatment, the morphological features of apoptosis were observed by phase-contrast microscopy. Cell survival was measured by MTT (3-(4,5-dimethylthiazol-2-yl)-2,5-diphenyltetrazolium bromide) assays and by flow cytometric analysis after propidium iodide (PI) staining. Bcl-2 expression and signaling pathways (Akt, extracellular signal-regulated kinase, p38, c-Jun N-terminal kinase) were examined with or without Hsp70 downregulation.

**Results:**

Hsp70 downregulation in RA FLSs, induced by small interfering RNA (siRNA), was confirmed by reverse transcriptase-polymerase chain reaction and Western blotting. When treated with SNP, Hsp70 downregulated cells showed markedly less cell blebbing, cytoplasmic condensation, and nuclear shrinkage than non-downregulated control cells. Furthermore, Hsp70 downregulated cells were found to survive better than control cells in MTT assays (mean of absorbance ratio, 4.39 in target cells versus 1.00 in control siRNA-treated cells versus 1.09 in lipofectamine-treated cells, *P *= 0.001) and according to PI staining results (mean M_1 _ratio, 0.21 in target cells versus 1.00 in control siRNA-treated cells versus 1.03 in lipofectamine-treated cells, *P *= 0.001). Bcl-2 expression and Akt phosphorylation were higher in Hsp70 downregulated RA FLSs than in control cells. When cells were treated with LY294002, a potent phosphoinositide 3-kinase inhibitor, Akt phosphorylation and Bcl-2 levels were reduced and Hsp70 downregulation no longer had a cytoprotective effect.

**Conclusions:**

Knock-down of Hsp70 protects RA FLSs from nitric oxide-induced apoptosis by activating the Akt signaling pathway. These results suggest that Hsp70 has a pro-apoptotic role in RA FLSs.

## Introduction

Rheumatoid arthritis (RA) is a chronic inflammatory disorder that involves mainly joint synovium. One of the major characteristics of RA synovium is the tumor-like growth of fibroblast-like synoviocytes (FLSs) that invade adjacent articular cartilage and bone [1]. Although the mechanism of FLS hyperplasia in RA is not fully understood, it is explained in part by excessive survival and/or anti-apoptotic signals to FLSs transmitted by inflammatory cells and cytokines. For example, it has been well established that tumor necrosis factor-alpha (TNF-α), a key cytokine in RA, activates genes that mediate proliferative and inflammatory responses [2]. Other anti-apoptotic apparatuses expressed in RA FLSs include FLIP (Fas-associated death domain-like interleukin [IL]-1β-converting enzyme-inhibitory protein) [3], sentrin [4], mutated p53 [5,6], and the activation of the nuclear factor-kappa-B or the Akt signaling pathways or both [7,8].

Heat shock protein 70 (Hsp70) is a molecular chaperone that is rapidly induced by physical and chemical stresses. The anti-apoptotic function of Hsp70 depends on its ability to interact with protein substrates that are not always associated with the chaperoning activity. The mechanisms by which Hsp70 exerts its anti-apoptotic function encompass the inhibition of the c-Jun N-terminal kinase (JNK) signaling pathway, caspase activation, mitochondrial cytochrome *c *release, and apoptosome formation [9]. Although the anti-apoptotic role of Hsp70 has been demonstrated in a number of studies in various cell types and under different conditions, several other studies have shown that the overexpression of Hsp70 promotes cell death, which suggests that Hsp70 has dual functionality depending on cell and stimulus type [10-12]. It has been reported that the expression of Hsp70 is higher in both tissue and cultured RA FLSs than in the FLSs of osteoarthritis and that inflammatory cytokines, such as TNF-α and IL-1β, that exist abundantly in RA joint fluid further increase Hsp70 expression in cultured RA FLSs [13]. However, no study has investigated the actual role of Hsp70 in the survival of RA FLSs. In this study, we investigate the effect of Hsp70 downregulation on RA FLS apoptosis induced by sodium nitroprusside (SNP), a nitric oxide (NO) donor.

## Materials and methods

### Primary culture of fibroblast-like synoviocyte derived from rheumatoid arthritis patient synovium

Synovium was obtained from the affected joints of five patients undergoing joint replacement surgery (Table 1). Individual age data were removed to protect the identities of patients. All patients met the 1987 American Rheumatism Association criteria for RA [14]. The isolation and culture of FLSs were performed as previously described [5]. Briefly, minced synovium was incubated with 1 mg/mL collagenase type VIII (Sigma-Aldrich, St. Louis, MO, USA) in serum-free RPMI 1640 medium (Gibco, now part of Invitrogen Corporation, Carlsbad, CA, USA) for 1 hour at 37°C, filtered, extensively washed, and cultured in Dulbecco's modified Eagle's medium (DMEM) (Invitrogen Corporation) supplemented with 10% fetal bovine serum (FBS) (Invitrogen Corporation). Cells were allowed to adhere overnight, and adherent FLSs were grown in DMEM containing 10% FBS. FLSs at passages 3 to 7 were used for the experiments. This study was approved by the Institutional Review Board of Seoul National University Hospital (#H-0603-134-170), and informed consent was obtained from participants.

**Table 1:** Clinical data of five patients with rheumatoid arthritis at the time of joint surgery ^a^

**Patient**	**Disease duration^b^, months**	**Site of surgery**	**Medication**
1	74	Knee	MTX 15 mg/wk, SSZ 1 g/d, Pd 5 mg/d
2	60	Knee	NSAIDs
3	97.5	Knee	Pd 10 mg/d
4	121	Knee	Pd 10 mg/d
5	180	Hip	HCQ 200 mg/d

^a^Age of the patients was 57.0 ± 15.6 years (age range 35 to 73 years), and they were all female. ^b^Disease duration is the time interval between symptom onset of rheumatoid arthritis and joint replacement surgery. HCQ, hydroxychlorquine; MTX, methotrexate; NSAID, nonsteroidal anti-inflammatory drug; Pd, prednisolone; SSZ, sulfasalazine.

### Cell treatments

Cells were incubated with TNF-α (R&D Systems, Minneapolis, MN, USA) at 10 ng/mL for 8 hours where indicated. To induce apoptosis, cells were treated with 1 mM SNP (Sigma-Aldrich) for 24 hours in a light-shielded state. LY294002 (Cell Signaling Technology, Inc., Danvers, MA, USA) at 2 to 10 μM was added to culture media to block phosphoinositide 3-kinase (PI3K) activity.

### Reverse transcriptase-polymerase chain reaction

Total mRNA was extracted from cultured RA FLSs, and single-stranded cDNA was synthesized from mRNA using reverse transcription kits (Intron Biotechnology, Seoul, Korea). The polymerase chain reaction (PCR) amplification of *hsp70 *was performed with an initial Taq (Invitrogen Corporation) activation at 95°C for 5 minutes followed by 25 amplification cycles of 15 seconds at 95°C, 30 seconds at 60°C, and 30 seconds at 60°C. The primers used were forward 5'-GGA-GGC-GGA-GAA-GTA-CAA-3' and reverse 5'-GCT-GAT-GAT-GGG-GTT-ACA-3' [15]. The semi-quantitative measurements were performed using *gapdh *(glyceraldehyde-3-phosphate dehydrogenase) as an internal reference.

### Western blotting

Total cell lysates were separated on 10% denaturating polyacrylamide gels and transferred to polyvinylidene difluoride membranes. The primary antibodies used were mouse monoclonal anti-Hsp70 antibody (donated by the Department of Biochemistry, Seoul National University College of Medicine, Korea), rat monoclonal anti-constitutional Hsp70 (Hsc70) antibody (StressGen Biotechnologies Corporation, Victoria, BC, Canada), rabbit monoclonal anti-Bcl-2 antibody (Santa Cruz Biotechnology, Inc., Santa Cruz, CA, USA), rabbit monoclonal anti-Akt/phospho-Akt, anti-extracellular signal-regulated kinase (ERK)/phospho-ERK, anti-c-Jun N-terminal kinase (JNK)/phospho-JNK, and anti-p38/phospho-p38 antibodies (Cell Signaling Technology, Inc.), and rabbit monoclonal anti-actin antibody (Sigma-Aldrich). The secondary antibodies used were horseradish peroxidase-conjugated anti-mouse IgG (The Jackson Laboratory, Bar Harbor, ME, USA), anti-rabbit IgG (The Jackson Laboratory), or anti-rat IgG (StressGen Biotechnologies Corporation) antibodies. Signals were developed using an ECL (enhanced chemiluminescence) system (Amersham Biosciences, now part of GE Healthcare, Little Chalfont, Buckinghamshire, UK).

### Transfection using small interfering RNA

FLSs were seeded at a density of 1.0 to approximately1.5 × 10^6^/mL and incubated overnight in antibiotics-free DMEM supplemented with 10% FBS. The following day, cells were washed with phosphate-buffered-saline, and then OPTI-MEM reduced serum medium (Invitrogen Corporation) was added to cells. Hsp70-specific small interfering RNA (siRNA) (forward 5'-CGA-CGG-AGA-CAA-GCC-CAA-GTT-3' and reverse 5'-CUU-GGG-CUU-GUC-UCC-GUC-GTT-3'; Invitrogen Corporation) [15] and a nucleic acid transferring agent, lipofectamine 2000 (Invitrogen Corporation), were incubated together in OPTI-MEM reduced serum medium for 15 minutes at room temperature to form an siRNA-lipofectamine complex. The siRNA-lipofectamine complex-containing medium was added to cells to a final siRNA concentration of 25 nM. Six hours later, the complex-containing medium was exchanged with antibiotics-free DMEM supplemented with 10% FBS. The second round of transfection was performed 48 hours after the first transfection, using the same method. Cells transfected twice with 25 nM control siRNA of medium GC content (Invitrogen Corporation) and cells transfected with lipofectamine only were used as controls.

### Assessment of cell viability and apoptosis

Morphological changes were observed after SNP treatment under a phase-contrast microscope (Olympus, Tokyo, Japan). In addition, 3-(4,5-dimethylthiazol-2-yl)-2,5-diphenyltetrazolium bromide (MTT) (Sigma-Aldrich) assays were performed to assess cell viability. Apoptotic cell fractions (sub-G_0_/G_1 _portions or M_1 _fractions) were measured by propidium iodide (PI) (Sigma-Aldrich) staining and flow cytometry (FACSCalibur; BD Biosciences, San Jose, CA, USA). The absorbance (measured at 570 nm) and apoptotic fraction were normalized to those of control siRNA-treated cells in each experiment.

### Statistical analysis

Continuous values were presented as mean ± standard deviation. Analysis of variance (ANOVA) was used to compare mean values of more than two groups. Two-tailed *P *values of less than 0.05 were considered significant. All statistical calculations were performed using SPSS version 12 (SPSS Inc., Chicago, IL, USA).

## Results

### Expression of Hsp70 in cultured rheumatoid arthritis fibroblast-like synoviocytes with or without tumor necrosis factor-alpha

**Figure 1:**
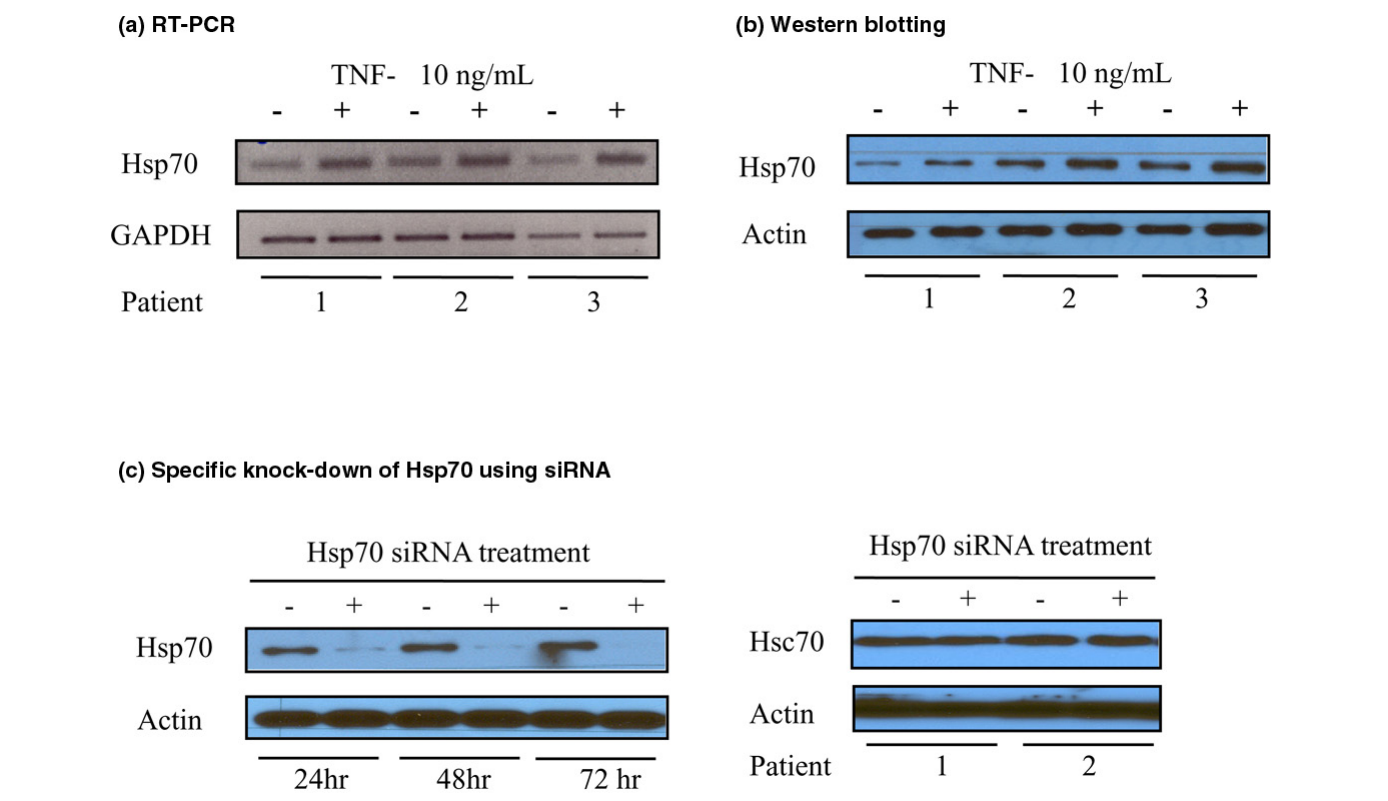
Expression and specific knock-down of heat shock protein 70 (Hsp70) in rheumatoid arthritis (RA) fibroblast-like synoviocytes (FLSs).  Hsp70 was expressed in cultured RA FLSs as shown by reverse transcriptase-polymerase chain reaction **(a) **and Western blotting **(b)**. Hsp70 levels were increased by tumor necrosis factor-alpha (TNF-α) treatment. Gene knock-down was specific for Hsp70; constitutional Hsp70 (Hsc70) levels were unaffected **(c)**. The experiments were performed in RA FLSs from five patients and these are representative figures. GAPDH, glyceraldehyde-3-phosphate dehydrogenase; siRNA, small interfering RNA.

Hsp70 expressions in cultured RA FLSs were confirmed by reverse transcriptase-PCR and Western blotting (Figure 1a, b). They were increased after 8 hours of treatment with 10 ng/mL TNF-α.

### Specific knock-down of Hsp70 expression in rheumatoid arthritis fibroblast-like synoviocyte

Targeted knock-down of Hsp70 by specific siRNA was observed in RA FLSs (Figure 1c). Because knock-down was incomplete after a single transfection, a second round of transfection was performed 48 hours after the first transfection. Almost complete knock-down of Hsp70 expression was then achieved and sustained for up to 72 hours. The expression of Hsc70 was unaffected (observed at 48 hours after the second transfection) by Hsp70-specific siRNA despite its 86% amino acid homology with Hsp70 (Figure 1c).

### Effect of Hsp70 knock-down on nitric oxide-induced apoptosis in rheumatoid arthritis fibroblast-like synoviocyte

**Figure 2:**
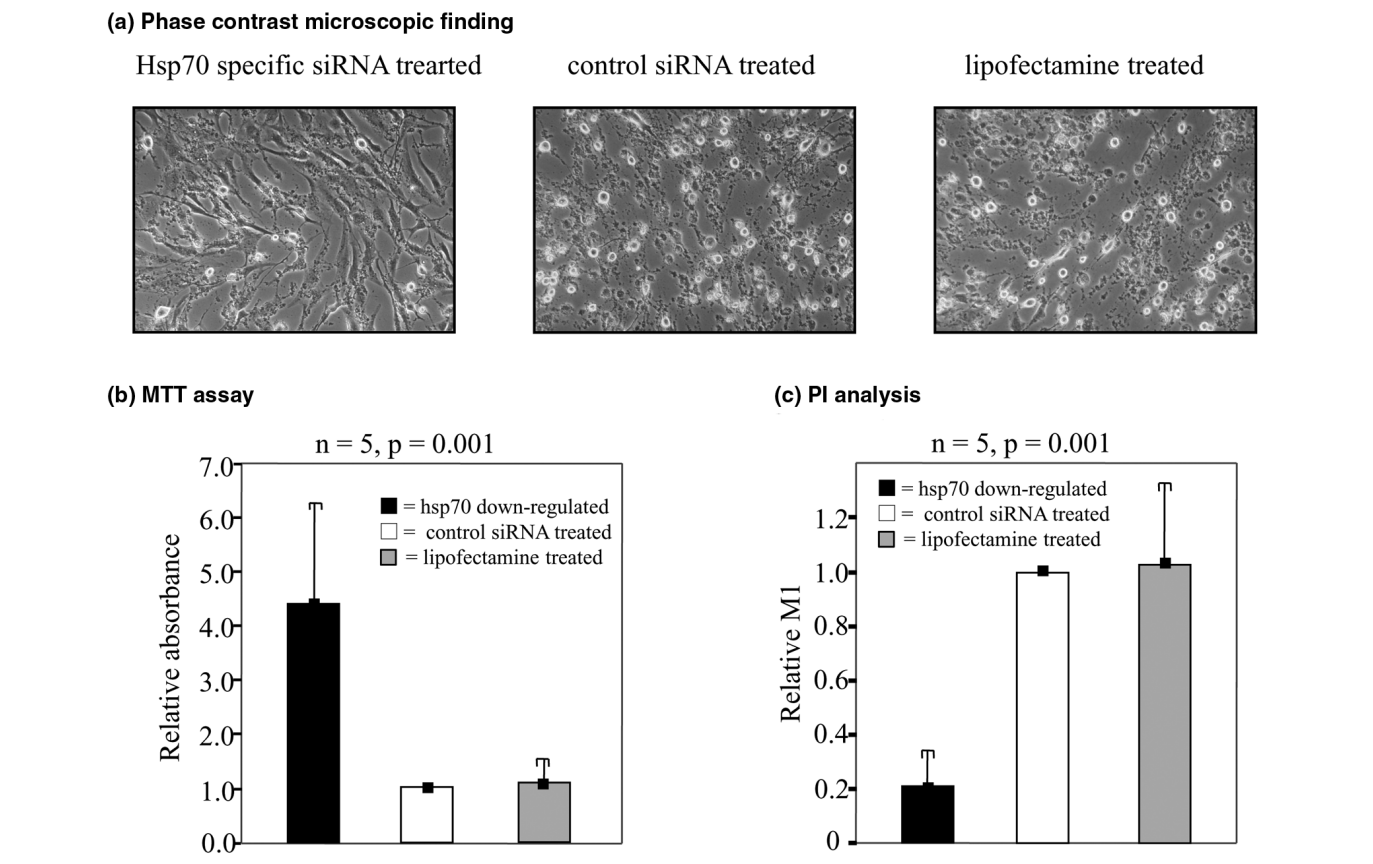
Effect of heat shock protein 70 (Hsp70) knock-down on nitric oxide-induced apoptosis in rheumatoid arthritis (RA) fibroblast-like synoviocytes (FLSs).  To induce apoptosis, cells were treated with 1 mM sodium nitroprusside (Sigma-Aldrich, St. Louis, MO, USA) for 24 hours in a light-shielded state. The cytoprotective effect of Hsp70 knock-down was observed by phase-contrast microscopy **(a)**. MTT assays showed that cell viability was significantly greater in Hsp70 downregulated FLSs (left) than in control FLSs (middle = control small interfering RNA [siRNA]-treated cells, right = lipofectamine-treated cells) (mean of relative absorbance, 4.39 ± 2.82 versus 1.00 ± 0.00 versus 1.09 ± 0.30, *P *= 0.001 by analysis of variance [ANOVA]) **(b)**. Propidium iodide (PI) analysis showed that the apoptotic fraction in Hsp70 downregulated FLSs (left) was significantly lower than in control FLSs (middle = control siRNA-treated cells, right = lipofectamine-treated cells) (mean of relative M_1_, 0.21 ± 0.16 versus 1.00 ± 0.00 versus 1.03 ± 0.36, *P *= 0.001 by ANOVA) **(c)**. All experiments were performed in triplicate using five RA FLS samples. The histograms and error bars indicate mean and standard deviation of relative absorbance, respectively. MTT, 3-(4,5-dimethylthiazol-2-yl)-2,5-diphenyltetrazolium bromide.

After treatment with SNP (1 mM) for 24 hours, cell blebbing and cytoplasmic condensation were markedly lower in Hsp70 downregulated RA FLSs than in control RA FLSs (Figure 2a). MTT assays showed that the cell viability of Hsp70 downregulated FLSs was significantly higher than that of control FLSs (mean of relative absorbance of 4.39 ± 1.82 in target cells versus 1.00 ± 0.00 in control siRNA-treated cells versus 1.09 ± 0.30 in lipofectamine-treated cells, *P *= 0.001) (Figure 2b). PI analysis also revealed significantly lower apoptotic fractions in Hsp70 downregulated FLSs than in control FLSs (mean of relative M_1 _of 0.21 ± 0.16 in target cells versus 1.00 ± 0.00 in control siRNA-treated cells versus 1.03 ± 0.36 in lipofectamine-treated cells, *P *= 0.001) (Figure 2c).

### Intracellular Bcl-2 levels and signaling pathways

**Figure 3:**
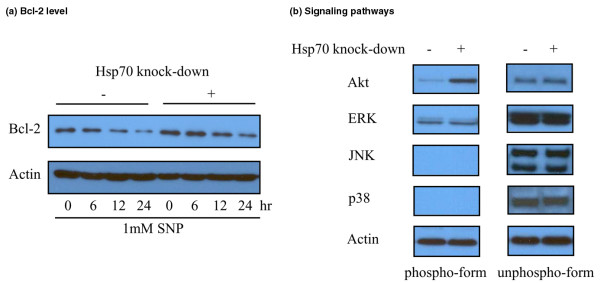
Bcl-2 level and signaling pathways in heat shock protein 70 (Hsp70) downregulated rheumatoid arthritis (RA) fibroblast-like synoviocytes (FLSs).  Levels of Bcl-2, a well-known inhibitor of nitric oxide-induced apoptosis, were increased by Hsp70 knock-down with or without sodium nitroprusside (SNP) treatment **(a)**. Western blotting showed that phospho-Akt levels were higher in Hsp70 downregulated RA FLSs than in controls **(b)**. The experiments were performed in five RA FLS samples and the representative figures are shown. ERK, extracellular signal-regulated kinase; JNK, c-Jun N-terminal kinase.

Because Bcl-2 is known to block NO-induced apoptosis [16,17], we investigated whether Hsp70 downregulation causes any change in cellular Bcl-2 level. Bcl-2 expression was higher in Hsp70 downregulated cells than in controls with or without SNP treatment (Figure 3a). We then explored the signaling pathways involved in the cytoprotective effect of Hsp70 downregulation. Western blotting revealed that Akt phosphorylation was higher in Hsp70 downregulated RA FLS than in controls. However, the ERK, p38, and JNK pathways were unaffected by Hsp70 downregulation (Figure 3b).

### Effect of Akt phosphorylation on the survival of rheumatoid arthritis fibroblast-like synoviocyte

**Figure 4:**
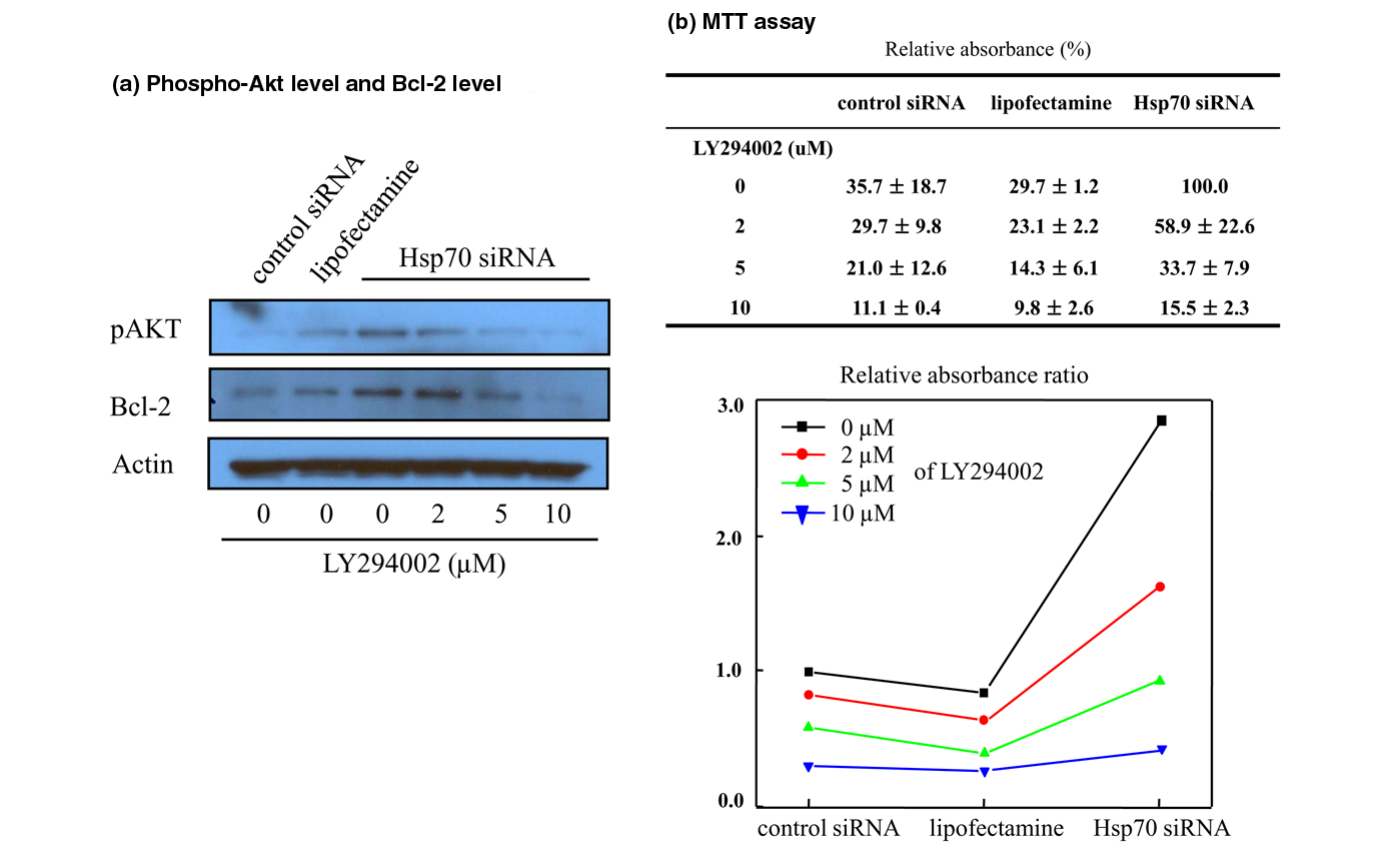
Effect of Akt phosphorylation on rheumatoid arthritis (RA) fibroblast-like synoviocyte (FLS) survival.  LY294002, a potent PI3K (phosphoinositide 3-kinase) inhibitor, decreased Akt phosphorylation in a dose-dependent manner, and Bcl-2 levels were found to be closely correlated with phospho-Akt levels **(a)**. When cells were treated with LY294002, the cytoprotective effect of heat shock protein 70 (Hsp70) knock-down disappeared **(b)**. The experiments were performed using three RA FLS samples (a, b) in triplicate (b). The representative figures are shown. MTT, 3-(4,5-dimethylthiazol-2-yl)-2,5-diphenyltetrazolium bromide; siRNA, small interfering RNA.

LY294002 (10 μM) decreased Akt phosphorylation to the control level, and this inhibition of Akt phosphorylation was LY294002 dose-dependent, which indicated that Akt is phosphorylated via PI3K (Figure 4a). In addition, Bcl-2 levels were found to be closely correlated with phospho-Akt levels (Figure 4a). Furthermore, Hsp70 downregulated cells did not exhibit resistance to SNP treatment when Akt signaling was inhibited, and this loss of Hsp70 downregulation-induced cytoprotection was dose-dependent of LY294002 (Figure 4b).

## Discussion

This study shows that Hsp70 is expressed in RA FLSs and that knock-down of Hsp70 protects RA FLSs from NO-induced apoptosis via the Akt signaling pathway. This result is consistent with that of Schett and colleagues [13], who found that RA FLSs express Hsp70 and that this expression is increased by inflammatory cytokines, such as TNF-α. On the other hand, Schick and colleagues [18] found that Hsc70, and not Hsp70, was overexpressed in RA synovial tissues and cultured FLSs. They suspected that anti-Hsp70 antibody in the study of Schett and colleagues might have cross-reacted with Hsc70. However, the specificity of the anti-Hsp70 antibody used in the present study has been characterized previously [19]. Furthermore, our observation that anti-Hsp70 antibody demonstrated Hsp70 downregulation by siRNA, whereas anti-Hsc70 antibody showed consistent Hsc70 expression, further supports specific antibody reactivity with Hsp70 in the present study.

Unexpectedly, we observed that Hsp70 downregulation protected RA FLSs from NO-induced apoptosis. This finding suggests that Hsp70 may be a pro-apoptotic protein in RA FLSs. Our results indicate that hsp70 interferes with Akt phosphorylation by binding an upstream protein of the Akt signaling pathway in RA FLSs. However, *in vivo*, abundant intracellular anti-apoptotic apparatuses generated by external survival/growth signals appear to overcome the negative effect of Hsp70 on Akt phosphorylation and on cell survival.

The anti-apoptotic effect mediated by Akt signaling pathway activation after Hsp70 knock-down in RA FLSs is consistent with the findings of the previous report, namely, that Akt phosphorylation is an important mechanism to protect RA FLSs from NO-induced apoptosis [20]. Akt phosphorylation has been shown to regulate Hsp70 expression [21,22]. However, to the best of our knowledge, this is the first report to suggest that Hsp70 is involved in the regulation of the Akt signaling pathway. However, the direct molecular mechanism by which Hsp70 knock-down promotes Akt phosphorylation remains to be determined.

Recently, anti-cytokine therapies for RA have entered the spotlight [23-25]. However, response to anti-cytokine agents is often only partial and some patients are refractory to these agents. Therefore, treatments with different mechanisms need to be combined. Although blocking anti-apoptotic proteins is a candidate strategy, the results of the present study do not support the notion that the downregulation of Hsp70 promotes the regression of hyperplastic synovium. Alternatively, the *in vivo *effect of Hsp70 knock-down might differ from that observed *in vitro*. Recently, the biological role of extracellular hsp70, particularly in terms of immune response, including the cross-presentation of antigenic peptide and maturation of antigen-presenting cells (APCs), was substantively updated [26-28]. Because the synovial fluid of patients with RA contains high levels of soluble Hsp70, which interacts with APCs via a plethora of surface receptors [29], the effect of Hsp70 knock-down is likely to be complex *in vivo*.

## Conclusions

In summary, this study shows that Hsp70 is expressed in RA FLSs and that knock-down of Hsp70 protects RA FLSs from NO-induced apoptosis by activating the Akt signaling pathway. These results suggest that Hsp70 has a pro-apoptotic role in RA FLS.

## Abbreviations

APC: antigen-presenting cell; DMEM: Dulbecco's modified Eagle's medium; ERK: extracellular signal-regulated kinase; FBS: fetal bovine serum; FLS: fibroblast-like synoviocyte; Hsc70: constitutional heat shock protein 70; Hsp70: heat shock protein 70; IL: interleukin; JNK: c-Jun N-terminal kinase; MTT: 3-(4,5-dimethylthiazol-2-yl)-2,5-diphenyltetrazolium bromide; NO: nitric oxide; PCR: polymerase chain reaction; PI: propidium iodide; PI3K: phosphoinositide 3-kinase; RA: rheumatoid arthritis; siRNA: small interfering RNA; SNP: sodium nitroprusside; TNF-α: tumor necrosis factor-alpha.

## Competing interests

The authors declare that they have no competing interests.

## Authors' contributions

EHK was involved in the conception and design of this study, carried out the cellular, molecular, and genetic studies, and drafted the manuscript. YWS was involved in the conception and design of this study and helped draft the manuscript. DJK, EYL, and YJL participated in the design of the study. EBL helped interpret the data. All authors have read and approved the final manuscript.
